# Rapid Discrimination of *Panax quinquefolium* and *Panax ginseng* Using the Proofman-Duplex-LMTIA Technique

**DOI:** 10.3390/molecules28196872

**Published:** 2023-09-29

**Authors:** Xiaodong Zhang, Zongding Li, Yaoxuan Zhang, Dandan Xu, Liang Zhang, Fugang Xiao, Deguo Wang

**Affiliations:** 1 Henan Key Laboratory of Biomarker Based Rapid-Detection Technology for Food Safety, Food and Pharmacy College, Xuchang University, Xuchang 461000, China; zxd95@xcu.edu.cn (X.Z.); 17603939318@163.com (Z.L.); 12017029@xcu.edu.cn (L.Z.); xfug@163.com (F.X.); 2 College of Grain and Food, Henan University of Technology, Zhengzhou 450001, China; zhangyxlll000@163.com; 3 School of Food and Biological Engineering, Henan University of Science and Technology, Luoyang 471000, China; X1050983354@163.com

**Keywords:** *Panax quinquefolium*, *Panax ginseng*, ladder melting temperature isothermal amplification, Proofman probe, rapid identification

## Abstract

This study aims to establish a rapid identification method based on the Proofman-LMTIA technique for distinguishing between *Panax quinquefolium* and *Panax ginseng*. By targeting specific 18S rDNA sequences, suitable primers and Proofman probes labeled FAM or JOE were designed for LMTIA. Initially, single-species-primer Proofman-LMTIA assays were performed separately for each ginseng type to optimize reaction temperature, assess sensitivity and specificity, and determine the detection limit. Subsequently, both sets of primers and their corresponding probes were combined in the same reaction system to further optimize reaction conditions, evaluate sensitivity, and assess stability. Finally, the developed Proofman-duplex-LMTIA technique was employed to detect *P. quinquefolium* and *P. ginseng* slices available in the market. Single-plex Proofman-LMTIA assays revealed that the optimal reaction temperature for both *P. quinquefolium* and *P. ginseng* was 62 °C. The sensitivity was as low as 1 pg/μL, with a detection limit of 0.1%, and both showed excellent specificity. The optimal temperature for Proofman-duplex-LMTIA assays was 58 °C. This method could simultaneously identify *P. quinquefolium* and *P. ginseng*. Testing 6 samples of *P. ginseng* and 11 samples of *P. quinquefolium* from the market resulted in a 100% positive rate for all samples. This study successfully established a rapid, simple, sensitive, and specific Proofman-duplex-LMTIA identification method for *P. quinquefolium* and *P. ginseng*. It provides an effective means for quality control of *P. quinquefolium*, *P. ginseng*, and related products.

## 1. Introduction

*Panax quinquefolium* L. belongs to the Araliaceae family, and its dried roots are known for their effects in replenishing Qi, nourishing Yin, clearing heat, and generating body fluids [1]. It is commonly used to treat symptoms such as Qi deficiency, Yin deficiency, heat fatigue, cough, asthma, and blood-streaked sputum [1]. Similarly, *Panax ginseng* C. A. Mey., also belonging to the Araliaceae family, utilizes its dried roots and rhizomes for various therapeutic purposes, including enhancing vital energy, stabilizing abnormal pulses, tonifying the spleen, invigorating the lungs, promoting blood production, nourishing fluids, and improving cognitive functions [1]. Nevertheless, due to commercial interests and their visual resemblance, *P. quinquefolium* and *P. ginseng* are often mistakenly identified and substituted in the market [2,3]. Additionally, their seeds are difficult to differentiate due to their similar appearance [4]. As a result, accurate and rapid differentiation of these two plants and their products is crucial for effective utilization and seed recognition.

Various identification methods and standards, such as morphological identification, microscopic examination, thin-layer chromatography of extracts, and primary ginsenoside content determination, have been established in the Chinese Pharmacopoeia to differentiate between *P. quinquefolium* and *P. ginseng* [1]. However, these methods exhibit significant limitations in practical applications. Researchers have been actively seeking more rapid and accurate detection methods to address these issues. Leveraging the relative stability of DNA, previous studies have developed several detection techniques, including conventional PCR [5], PCR-based restriction fragment length polymorphism [6], single nucleotide polymorphism (SNP) -based methods [3] and multiplex PCR [7]. While these methods partially resolved the identification challenge, they still present challenges, such as prolonged processing time, high costs, and intricate operations. To overcome these limitations, we introduce the Proofman-Ladder Melting Temperature Isothermal Amplification (Proofman-LMTIA) technique.

LMTIA is a nucleic acid rapid detection technique based on the isothermal amplification of a ladder-like melting curve [8]. Compared to traditional PCR, LMTIA offers several advantages: (1) it does not rely on expensive PCR or qPCR instruments, employing isothermal amplification; (2) rapid reaction, with each reaction taking only 30 min; (3) suitable for shorter target sequences, requiring a minimum length of 60 bp for amplification; (4) high sensitivity, with studies showing 50 times higher sensitivity compared to Loop-mediated isothermal amplification (LAMP) technology [9]; and (5) broad applicability, as the employed *Bst* enzyme possesses reverse transcriptase activity, making it suitable for various nucleic acid sequences, including RNA. This technique has been successfully applied in diverse fields, such as the rapid detection of cassava components in sweet potato starch [10] and African swine fever virus detection [11].

LMTIA generates different DNA structures during amplification [8]. To realize sequence-specific detection, we add the Proofman probes, labeled with a fluorophore and a quencher at its 3′ and 5′ ends, respectively, and a thermostable DNA polymerase *pfu*. A strategic 3′ end mismatch can activate *pfu*’s 3′→5′ exonuclease activity. When the probe binds to the target, *pfu* cleaves the mismatched nucleotide, releasing the fluorophore. The cleaved probe acts as a primer for amplification. Target fragment(s) presence in positive samples leads to green (FAM) and/or yellow (JOE) fluorescence, while negative samples lack amplification and fluorescence. LMTIA product detection relies on increasing fluorescence during amplification via the Proofman probe(s), enhancing accuracy through sequence-specific binding and cleavage [12]. This Proofman-LMTIA technique has been successfully applied in the rapid detection of harmful bacteria *Listeria monocytogenes* contamination in food [12] and soy-derived components in dairy products [13].

The Proofman-LMTIA technique integrates Proofman probes into LMTIA reactions to simultaneously detect multiple species. In this study, focusing on the 18S rDNA sequence differences between *P. quinquefolium* and *P. ginseng*, we designed specific primers and optimized the Proofman-LMTIA technique for amplification temperature, sensitivity, and specificity. Furthermore, we combined primers and fluorescent probes for both ginseng species and optimized the reaction system. Ultimately, we applied the optimized method to the detection of commercially available ginseng slice samples, offering an effective solution for the authenticity verification of *P. quinquefolium* and *P. ginseng* materials, seeds, foods, and medicines.

## 2. Results

### 2.1. Sequence Analysis and Primer Design for 18S rDNA of P. quinquefolium and P. ginseng

**Figure 1:**
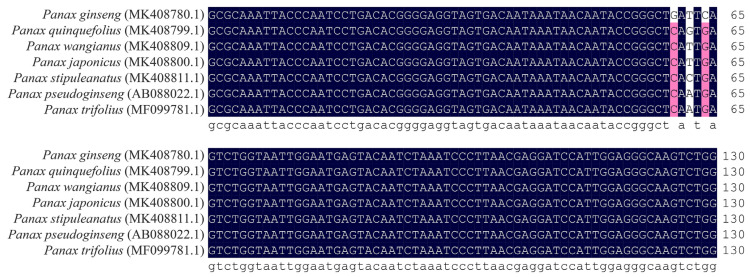
Alignment results of 18S rDNA differential sequences among *P. quinquefolium*, *P. ginseng* and their relatives.

The 18S rDNA sequences of *P. quinquefolium* and *P. ginseng* were aligned using DNAMAN v7.0.2.176 software [14]. The alignment revealed the presence of three SNP sites between *P. quinquefolium* and *P. ginseng*, occurring at positions 60 (G/C), 62 (T/G), and 64 (C/G) (Figure 1). Following the principles of LMTIA primer design, primers were designed for both *P. quinquefolium* and *P. ginseng*, along with *P. ginseng*-specific probe Shen-R-LF and *P. quinquefolium*-specific probe Shen-X-LF (Table 1, Figure S1).

### 2.2. Optimization of Single-plex Proofman-LMTIA Reaction Temperature for P. quinquefolium and P. ginseng

**Figure 2:**
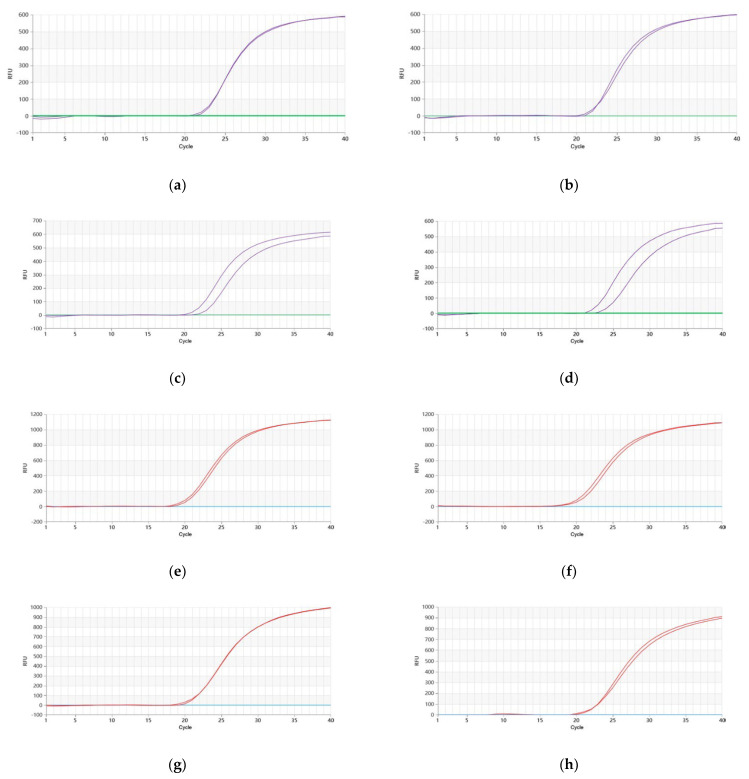
Proofman−LMTIA reaction for *P. quinquefolium* and *P. ginseng* at different temperatures. (**a**–**d**): Proofman−LMTIA reaction for *P. quinquefolium* at 61 °C, 62 °C, 63 °C, and 64 °C, respectively. Purple curve: genomic DNA of *P. quinquefolium*; green curve: DEPC−treated water. (**e**–**h**): Proofman-LMTIA reaction for *P. ginseng* at 61 °C, 62 °C, 63 °C, and 64 °C, respectively. Red curve: genomic DNA of *P. ginseng*; blue curve: DEPC−treated water.

The results indicated that when using *P. quinquefolium* genomic DNA as the template, the Proofman-LMTIA reaction performed well at temperatures of 61 °C, 62 °C, 63 °C, and 64 °C. The negative control with DEPC-treated water showed no amplification, while *P. quinquefolium* genomic DNA exhibited amplification at all temperatures tested. Notably, the most efficient amplification was observed at 62 °C, beginning around the 21st cycle (Figure 2a–d). Similarly, when utilizing *P. ginseng* genomic DNA as the template, the LMTIA reaction demonstrated optimal performance at temperatures of 61 °C, 62 °C, 63 °C, and 64 °C. Again, the negative control showed no amplification, whereas *P. ginseng* genomic DNA exhibited successful amplification. The most efficient amplification was achieved at 62 °C, starting approximately at the 17th cycle (Figure 2e–h). Hence, 62 °C was chosen as the optimal reaction temperature for single-plex Proofman-LMTIA reactions for both *P. quinquefolium* and *P. ginseng*.

### 2.3. Single-Plex Proofma-LMTIA Sensitivity Assay for P. quinquefolium and P. ginseng

**Figure 3:**
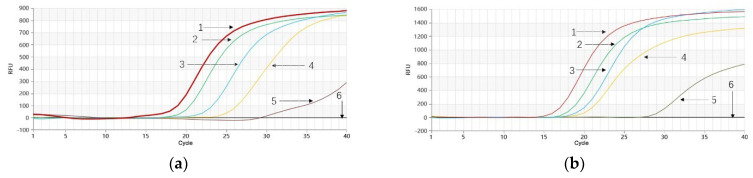
Proofman−LMTIA reaction for *P. quinquefolium* and *P. ginseng* at different concentration of template. (**a**) 1–5: genomic DNA concentration of *P. quinquefolium* was 10 ng/μL, 1 ng/μL, 100 pg/μL, 10 pg/μL and 1 pg/μL. 6: DEPC−treated water. (**b**) 1–5: genomic DNA concentration of *P. ginseng* was 10 ng/μL, 1 ng/μL, 100 pg/μL, 10 pg/μL, and 1 pg/μL. 6: DEPC−treated water.

A gradient dilution of *P. quinquefolium* genomic DNA was subjected to Proofman-LMTIA reactions. The results revealed successful amplification for DNA concentrations of 10 ng/μL, 1 ng/μL, 100 pg/μL, and 10 pg/μL. Even DNA at a concentration of 1 pg/μL exhibited amplification, while the negative control with DEPC-treated water showed no amplification curve (Figure 3a). Therefore, the sensitivity of the Proofman-LMTIA reaction for *P. quinquefolium* was determined to be 1 pg/μL. Similar sensitivity results were obtained for *P. ginseng* genomic DNA dilutions, where concentrations of 10 ng/μL, 1 ng/μL, 100 pg/μL, 10 pg/μL, and 1 pg/μL all exhibited successful amplification. The negative control with DEPC-treated water displayed no amplification, indicating a sensitivity of 1 pg/μL for the Proofman-LMTIA reaction for *P. ginseng* (Figure 3b).

### 2.4. Single-Plex Proofman-LMTIA Specificity Test for P. quinquefolium and P. ginseng

**Figure 4:**
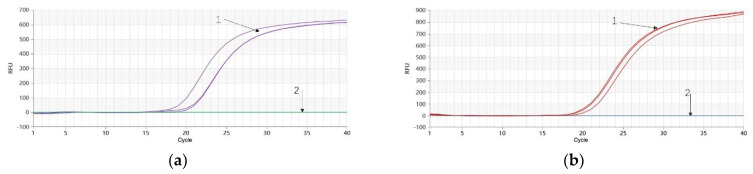
Proofman−LMTIA specific reaction for *P. quinquefolium* and *P. ginseng*. (**a**) 1: 10 ng/μL of genomic DNA of *P. quinquefolium*; 2: 10 ng/μL of genomic DNA of *P. ginseng*; (**b**) 1: 10 ng/μL of genomic DNA of *P. ginseng*; 2: 10 ng/μL of genomic DNA of *P. quinquefolium*.

The outcomes demonstrated that the Proofman-LMTIA reaction using 10 ng/μL of *P. quinquefolium* genomic DNA as the template resulted in successful amplification, while the LMTIA reaction using 10 ng/μL of *P. ginseng* genomic DNA did not amplify (Figure 4a). This indicates good primer specificity for *P. quinquefolium*. Conversely, in the *P. ginseng* Proofman-LMTIA reaction, using 10 ng/μL of *P. ginseng* genomic DNA as the template produced successful amplification, while 10 ng/μL of *P. quinquefolium* genomic DNA did not amplify (Figure 4b), signifying favorable primer specificity for *P. ginseng*.

### 2.5. Single-Plex Simulated Adulteration Test for P. quinquefolium and P. ginseng

**Figure 5:**
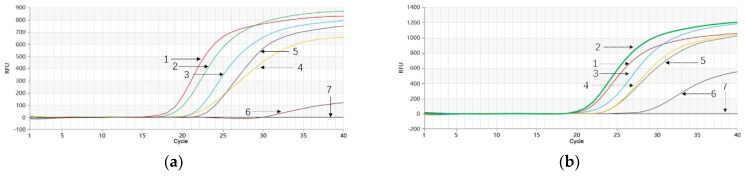
Proofman−LMTIA reaction for simulated adulteration experiment of *P. quinquefolium* and *P. ginseng*. (**a**) 1–6: 100%, 20%, 10%, 5%, 1%, and 0.1% of genomic DNA of *P. quinquefolium* in the mixture of *P. quinquefolium* and *P. ginseng*; 7: DEPC−treated water; (**b**) 1–6: 100%, 20%, 10%, 5%, 1%, and 0.1% of genomic DNA of *P. ginseng* in the mixture of *P. quinquefolium* and *P. ginseng*; 7: DEPC−treated water.

As depicted in Figure 5a, *P. quinquefolium* genomic DNA samples prepared at volumetric proportions of 100.0%, 20.0%, 10.0%, 5.0%, 1.0%, and 0.1% in a mixture with *P. ginseng* genomic DNA exhibited successful amplification for the first five samples. Notably, the 0.1% sample also began amplifying around the 30th cycle (Figure 5a), indicating a detection limit of 0.1% for the *P. quinquefolium* Proofman-LMTIA reaction. Similarly, when *P. ginseng* genomic DNA samples were prepared at volumetric proportions of 100.0%, 20.0%, 10.0%, 5.0%, 1.0%, and 0.1% in a mixture with *P. quinquefolium* genomic DNA, successful amplification was observed for the first five samples (Figure 5b). Moreover, the 0.1% sample began amplification around the 27th cycle (Figure 5b), indicating a detection limit of 0.1% for the *P. ginseng* Proofman-LMTIA reaction.

### 2.6. Optimization of Proofman-Duplex-LMTIA Reaction Temperature for P. quinquefolium and P. ginseng

**Figure 6:**
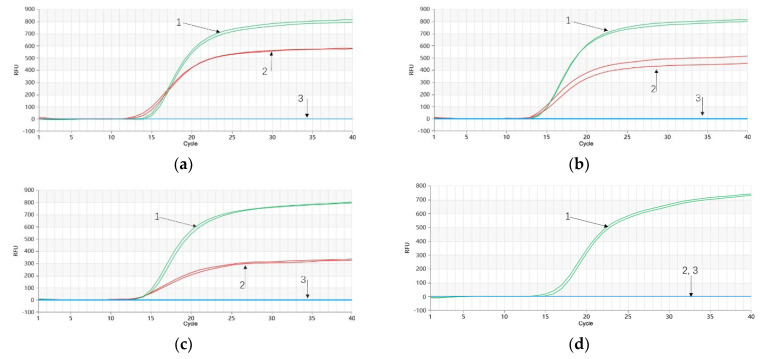
Proofman−duplex−LMTIA temperature optimization reaction for simultaneous detection of *P. quinquefolium* and *P. ginseng*. (**a**) 58 °C; (**b**) 60 °C; (**c**) 62 °C; and (**d**) 64 °C. 1: genomic DNA of *P. ginseng*; 2: genomic DNA of *P. quinquefolium*; 3: DEPC−treated water.

Temperature optimization for the Proofman-duplex-LMTIA reaction of *P. quinquefolium* and *P. ginseng* was conducted at 58 °C, 60 °C, 62 °C, and 64 °C. The results, as shown in Figure 6, demonstrated efficient amplification and reproducibility for both *P. quinquefolium* and *P. ginseng* genomic DNA at temperatures of 58 °C, 60 °C, and 62 °C. However, at 64 °C, only *P. ginseng* genomic DNA exhibited amplification, while *P. quinquefolium* genomic DNA did not amplify (Figure 6d). Since these temperatures had minimal impact on the *P. ginseng* Proofman-duplex-LMTIA reaction, and the highest amplification efficiency for *P. quinquefolium* genomic DNA was observed at 58 °C (Figure 6a), 58 °C was chosen as the optimal temperature for the Proofman-duplex-LMTIA detection of both *P. quinquefolium* and *P. ginseng*.

### 2.7. Sensitivity Test for Proofman-Duplex-LMTIA Detection of P. quinquefolium and P. ginseng

**Figure 7:**
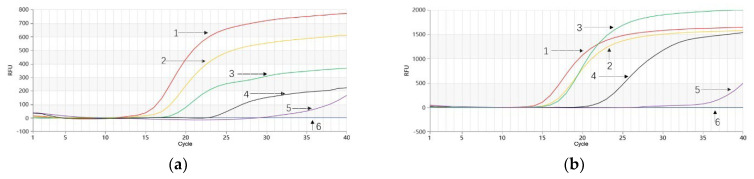
Proofman−duplex−LMTIA sensitivity detection for simultaneous detection of *P. quinquefolium* and *P ginseng*. (**a**) Proofman-duplex-LMTIA sensitivity detection for *P. quinquefolium*. 1–5: genomic DNA concentration of *P. quinquefolium* was 10 ng/μL, 1 ng/μL, 100 pg/μL, 10 pg/μL, and 1 pg/μL, respectively. 6: DEPC−treated water. (**b**) Proofman-duplex-LMTIA sensitivity detection for *P. ginseng*. 1–5: genomic DNA concentration of *P. quinquefolium* was 10 ng/μL, 1 ng/μL, 100 pg/μL, 10 pg/μL, and 1 pg/μL, respectively. 6: DEPC−treated water.

Using the optimized reaction conditions, a sensitivity test was performed on gradient-diluted *P. quinquefolium* and *P. ginseng* genomic DNA samples for Proofman-duplex-LMTIA detection. The results, depicted in Figure 7, showed successful amplification for both *P. quinquefolium* and *P. ginseng* genomic DNA at concentrations of 10 ng/μL, 1 ng/μL, 100 pg/μL, 10 pg/μL, and 1 pg/μL within 40 reaction cycles (30 min). The negative control with DEPC-treated water exhibited no amplification in all cases (Figure 7a,b). This indicated that the sensitivity for Proofman-duplex-LMTIA detection of *P. quinquefolium* and *P. ginseng* genomic DNA could reach 1 pg/μL.

### 2.8. Stability Test for Proofman-Duplex-LMTIA Detection of P. quinquefolium and P. ginseng

**Figure 8:**
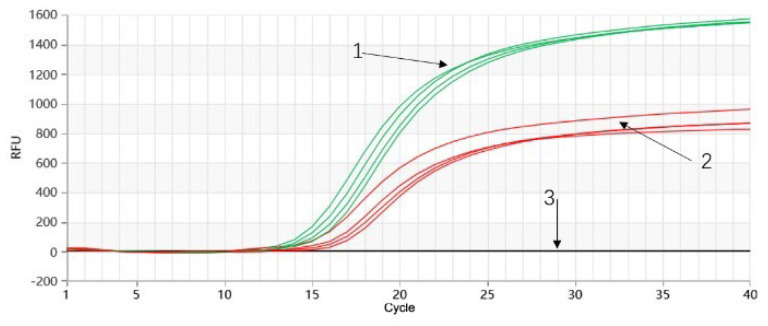
Proofman−duplex−LMTIA stability detection for simultaneous detection of *P. quinquefolium* and *P. ginseng*. 1: genomic DNA of *P. ginseng*; 2: genomic DNA of *P. quinquefolium*; 3: DEPC−treated water.

The Proofman-duplex-LMTIA method established in this study was tested in different laboratories, at different times, and under the operation of various experimenters. Using DEPC-treated water as the negative control and *P. quinquefolium* and *P. ginseng* genomic DNA as the positive control, four parallel samples were set for each. The detection rate of the positive control group in the Proofman-duplex-LMTIA detection system for *P. quinquefolium* and *P. ginseng* was 100%, while the negative control group with DEPC-treated water showed no amplification (Figure 8). This indicated the high stability of the Proofman-duplex-LMTIA detection system established in this study.

### 2.9. Results of Proofman-Duplex-LMTIA Detection of Marketed Slices of P. quinquefolium and P. ginseng

**Figure 9:**
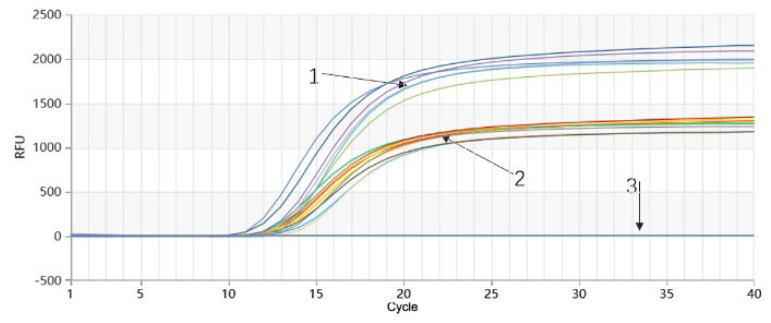
Simultaneously testing *P. quinquefolium* and *P. ginseng* slices in the market using Proofman−duplex−LMTIA. 1: genomic DNA of six *P. ginseng* slices; 2: genomic DNA of eleven *P. quinquefolium* slices; and 3: DEPC−treated water.

The Proofman-duplex-LMTIA detection method established in this study was applied to test 11 samples of *P. quinquefolium* slices and 6 samples of *P. ginseng* slices available on the market. The results showed that all 6 samples of *P. ginseng* slices and 11 samples of *P. quinquefolium* slices were positive, achieving a detection positivity rate of 100% (Figure 9).

## 3. Discussion

Both *P. quinquefolium* and *P. ginseng* are valuable traditional Chinese medicinal herbs with similar biological active components and morphological characteristics; yet, they hold distinct therapeutic values [3,15]. To differentiate between these two plants, the Chinese Pharmacopoeia has established methods and standards for identification, including visual examination, microscopic analysis, silica gel plate testing of extracts, and quantification of major ginsenosides [1]. However, these methods possess inherent limitations during implementation. For instance, visual examination requires considerable identification experience, is influenced by subjective factors, and becomes impractical when raw materials are ground into fine powders, processed into products, or used as nutritional supplements. Silica gel plate testing demands authentic herbal materials for reference, involves intricate procedures, and consumes extended time periods. Microscopic and high-performance liquid chromatography (HPLC) analyses require expensive specialized equipment and involve complex operations, leading to elevated costs.

DNA barcoding, a molecular identification approach, utilizes standardized, relatively short DNA sequences from the genomes of medicinal plants for authenticity assessment, and it has found widespread application in the authentication of traditional Chinese medicinal herbs and processed products [16]. Chen et al. established a DNA barcoding identification system for herbal medicines based on a combination of the ITS2 and psbA-trnH regions after extensive experimental research [14]. Despite its merits, this method still relies on costly PCR instruments and DNA sequencing technology, rendering broad adoption challenging.

This study develops a rapid and accurate method based on Proofman-duplex-LMTIA technology for distinguishing and detecting *P. quinquefolium* and *P. ginseng*, as well as their products. By introducing this innovative technology, we addressed the limitations of conventional identification methods, such as prolonged timeframes, high costs, and intricate procedures. Experimental outcomes underscore that the Proofman-duplex-LMTIA technology enables rapid identification and detection of *P. quinquefolium* and *P. ginseng*. Firstly, the design of species-specific primers was accomplished, ensuring the specificity of the primers by targeting the differences in the 18S rDNA sequence. Subsequently, the optimization of reaction temperature and sensitivity in LMTIA was achieved, facilitating the generation of discernible amplification signals even from samples containing minimal concentrations of genomic DNA under favorable conditions. Additionally, by mixing primers and fluorescent probes, coupled with reaction system optimization, simultaneous detection of both *P. quinquefolium* and *P. ginseng* was successfully achieved.

The Proofman-duplex-LMTIA technology showcased numerous advantages in this study, rendering it valuable for the differentiation of *P. quinquefolium* and *P. ginseng*: (1) Swift operation: with each reaction completed in just less than 30 min, the technology offers a faster alternative compared to traditional PCR methods, which is suitable for high-throughput sample testing. (2) High sensitivity: experimental results demonstrate the capacity of the technology to detect extremely low concentrations of genomic DNA, with sensitivity as low as 1 pg/μL, particularly useful for samples with limited DNA content. (3) Strong specificity: the introduction of Proofman probes and species-specific primers ensures specific detection of target species, reducing false-positive rates. (4) Simplicity of operation: in contrast to traditional PCR techniques, Proofman-duplex-LMTIA technology requires no complex instrumentation, making the operational process simpler. (5) Broad applicability: due to the reverse transcriptase activity of *Bst* enzyme, the technology is even applicable to the detection of RNA sequences.

The Proofman-duplex-LMTIA technology developed in this study holds considerable potential for various applications: (1) Herbal authentication: in the herbal market where misidentification and substitution of *P. quinquefolium* and *P. ginseng* occur, this technology can aid the herbal industry in ensuring the authenticity of used raw materials. (2) Seed identification: given the similarity of *P. quinquefolium* and *P. ginseng* seeds, making them challenging to differentiate, this technology can provide accurate identification methods in seed production and trading. (3) Quality control of food and pharmaceuticals: in ingredient identification for food, dietary supplements, and pharmaceuticals, the technology ensures the compliance of product constituents with standards. (4) Market supervision: regulatory agencies can utilize this technology to monitor the compliance and authenticity of products within the market.

In conclusion, this study successfully developed a method based on Proofman-duplex-LMTIA technology for the differentiation and detection of *P. quinquefolium* and *P. ginseng*, as well as their products. This innovative technology holds significant potential across sectors, including the herbal industry, food production, and market regulation. Future research could further explore the application of this technology in identifying other plant, animal, and microbe species, as well as its effectiveness in broader practical scenarios.

## 4. Materials and Methods

### 4.1. Materials

The standard decoction pieces of *P. quinquefolium* (pq-S) and *P. ginseng* (pg-S) were manufactured by Yuzhou Houshengtang Traditional Chinese Medicine Co., Ltd. in Henan, China; Six commercially available *P. ginseng* slices (pg1–pq6) and eleven commercially available *P. quinquefolium* slices (pq1-pq11) were purchased from Henan Dazhongyuan Pharmaceutical Co., Ltd. and Henan Zhangzhongjing Pharmacy Co., Ltd. (Table 2). 

To provide further details, pg1-pg3 slices were manufactured by Henan Lvhe Pharmaceutical Co., Ltd in Henan, China, while pg4-pg6 slices were produced by Bozhou Zhang Zhongjing Traditional Chinese Medicine Slices Co., Ltd in Anhui, China. For the *P. quinquefolium* slices, pq1-pg2 were manufactured by Jilin Tianjun Pharmaceutical Co., Ltd in Jilin, China, and pq3-pg7 slices were produced by Bozhou Zhang Zhongjing Traditional Chinese Medicine Slices Co., Ltd in Anhui, China. Moreover, pq8-pg9 slices were manufactured by Dongfanghong American Ginseng Pharmaceutical (Tonghua) Co., Ltd in Jilin, China, and pq10-pg11 slices were produced by Yuzhou Houshengtang Traditional Chinese Medicine Co., Ltd. in Henan, China.

### 4.2. Methods

#### 4.2.1. LMTIA Primer Design

The 18S rDNA sequences of *P. quinquefolium* (MK408799.1), *P. ginseng* (MK408780.1), *Panax wangianus* (MK408809.1), *Panax japonicus* (MK408800.1), *Panax stipuleanatus* (MK408811.1), *Panax pseudoginseng* (AB088022.1) and *Panax trifolius* (MF099781.1) were obtained from the GenBank database of NCBI [17]. DNAMAN v7.0.2.176 software was utilized to align these sequences to identify differing sites. The Oligo v7.56 software (Molecular Biology Insights, Inc., Colorado Springs, CO, USA) was employed to analyze and select sequences displaying a ladder-like melting temperature (Tm) structure. Using these sequences as the target and according to LMTIA primer design criteria [8], primers were designed with the online software Primer3Plus (https://www.primer3plus.com/, accessed on 1 June 2023).

#### 4.2.2. DNA Extraction

Herbal slices were ground into powder using liquid nitrogen. Exactly 100 mg of the powder was used for genomic DNA extraction according to the instructions of the Plant Genomic DNA Extraction Kit (DP305, Tiangen Biotech Co. Ltd., Beijing, China). The concentration and purity of genomic DNA were determined using a NanoDrop One spectrophotometer (Thermo Fisher Scientific, Waltham, MA, USA), with an A_260_/A_280_ ratio of 1.6–2.0 considered suitable for LMTIA detection. Extracted genomic DNA was stored at −20 °C.

#### 4.2.3. Reaction System Determination

A reaction system of 10 μL (0.16 μL of primer Shen-F1, 0.16 μL of primer Shen-B1, 0.04 μL of primer Shen-R-LF or Shen-X-LF, 0.04 μL of primer Shen-LB, 0.4 μL of Proofman probe, 2 μL of 5 × premix, 0.4 μL of *Bst* and *pfu* DNA Polymerase (Merit Biotech Shandong Co., Ltd., Heze, China), 2 μL of genomic DNA template, 4.8 μL of DEPC-treated water) was used for Proofman-LMTIA detection.

#### 4.2.4. Temperature Optimization Experiment

Using the established Proofman-LMTIA reaction system, separate experiments were conducted for *P. quinquefolium* and *P. ginseng* genomic DNA. DEPC-treated water was used as a negative control with two replicates for each reaction. Isothermal amplification was performed using the Gentier 96E fully automated medical PCR analysis system (Tianlong, Xi’an, China). Temperature gradients were set at 61 °C, 62 °C, 63 °C, and 64 °C, with fluorescence signal collection every 45 s. A total of 40 cycles were collected, with each experimental group repeated three times to minimize the influence of various factors on error.

#### 4.2.5. Temperature Sensitivity Determination Experiment

Using a gradient dilution method, the extracted genomic DNA of *P. quinquefolium* and *P. ginseng* was diluted to concentrations of 10 ng/μL, 1 ng/μL, 100 pg/μL, 10 pg/μL, and 1 pg/μL. These diluted samples were added to the optimized Proofman-LMTIA reaction system. DEPC-treated water was used as a negative control, and two parallel samples were set for each reaction. Fluorescence signals were collected every 45 s over 40 cycles. Each experimental group was repeated three times to ascertain the optimal reaction results.

#### 4.2.6. Specificity Experiment

For the specificity testing of *P. quinquefolium* primers, the Proofman-LMTIA reaction was conducted with 10 ng/μL of *P. quinquefolium* genomic DNA as the experimental group and 10 ng/μL of *P. ginseng* genomic DNA as the control group. Each experiment was performed with three parallel samples. The results were analyzed using the Gentier 96E fully automated medical PCR analysis system. Similarly, for the specificity testing of *P. ginseng* primers, the Proofman-LMTIA reaction was performed with 10 ng/μL of *P. ginseng* genomic DNA as the experimental group and 10 ng/μL of *P. quinquefolium* genomic DNA as the control group.

#### 4.2.7. Simulated Adulteration Experiment

Experiment simulating *P. quinquefolium* adulteration in *P. ginseng*: undiluted *P. quinquefolium* genomic DNA was mixed with *P. ginseng* DNA at varying proportions (*P. quinquefolium*: *P. ginseng*, *v*/*v*) of 0%, 0.1%, 1.0%, 5.0%, 10.0%, 20.0%, and 100.0%. The mixtures were added to the optimized Proofman-LMTIA reaction system, with DEPC-treated water as the negative control. Each experiment was repeated twice, and the amplification results were observed. Experiment simulating *P. ginseng* adulteration in *P. quinquefolium*: similar mixtures were created using varying proportions (*P. ginseng*: *P. quinquefolium*, *v*/*v*) of 0%, 0.1%, 1.0%, 5.0%, 10.0%, 20.0%, and 100.0%. These mixtures were added to the optimized Proofman-LMTIA reaction system, with DEPC-treated water as the negative control. Each experiment was repeated twice, and the amplification results were observed.

#### 4.2.8. Temperature Optimization Experiment for Simultaneous Detection of *P. quinquefolium* and *P. ginseng* Using Proofman-Duplex-LMTIA

Based on the laboratory’s experience in optimizing the Proofman-LMTIA system, a reaction system of 10 μL (0.16 μL of primer Shen-R-F1, 0.16 μL of primer Shen-R-B1, 0.04 μL of primer Shen-R-LF, 0.04 μL of primer Shen-R-LB, 0.16 μL of primer Shen-X-F2, 0.16 μL of primer Shen-X-B2, 0.04 μL of primer Shen-X-LF, 0.04 μL of primer Shen-X-LB, 0.4 μL of Proofman-R, 0.4 μL of Proofman-X, 2 μL of the 5 × premix, 0.4 μL of *Bst* and *pfu* DNA Polymerase, and 2 μL of genomic DNA template, 4 μL of DEPC-treated water) was used for detection. Considering that the optimal reaction temperature for single-plex LMTIA detection of *P. quinquefolium* and *P. ginseng* was 62 °C, the temperature screening for simultaneous detection was set at 58 °C, 60 °C, 62 °C, and 64 °C. *P. ginseng* and *P. quinquefolium* were used as positive controls at 1 ng/μL, and DEPC-treated water was the negative control. Fluorescence signals were collected every 45 s over 40 cycles.

#### 4.2.9. Sensitivity Testing Experiment for Simultaneous Detection of *P. quinquefolium* and *P. ginseng* Using Proofman-Duplex-LMTIA

Diluted genomic DNA of *P. quinquefolium* and *P. ginseng* at concentrations of 10 ng/μL, 1 ng/μL, 100 pg/μL, 10 pg/μL, and 1 pg/μL was added to the reaction system for detection. Each experiment was conducted with two parallel samples, and the process was repeated three times to assess the sensitivity of the developed method.

#### 4.2.10. Stability Experiment for Simultaneous Detection of *P. quinquefolium* and *P. ginseng* Using Proofman-Duplex-LMTIA

Utilizing the established Proofman-duplex-LMTIA detection technology for *P. quinquefolium* and *P. ginseng*, the detection was performed across different laboratories and at different times by randomly selecting an experimenter. Genomic DNA of *P. quinquefolium* and *P. ginseng* at 10 ng/μL served as positive controls, while DEPC-treated water was the negative control. Each experiment was conducted with four parallel samples, and the results were recorded to evaluate the amplification rate of negative samples and the reproducibility of positive samples.

#### 4.2.11. Proofman-Duplex-LMTIA Detection of Market *P. quinquefolium* and *P. ginseng* Slices

Six samples of *P. ginseng* and eleven samples of *P. quinquefolium* from the market were subjected to liquid nitrogen grinding and genomic DNA extraction. The Proofman-duplex-LMTIA detection method developed in this study was then applied for testing.

#### 4.2.12. Data Processing

The Gentier 96E fully automated medical PCR analysis system was used for analyzing the detection results and obtaining amplification curve charts. Data analysis and processing were further carried out using Microsoft Office 2019′s Excel v1808 and PowerPoint v1808 software.

## Figures and Tables

**Table 1:** Proofman-LMITA primers for *P. quinquefolium* and *P. ginseng*.

Primer Name	Sequence (5′ to 3′)
Shen-F1	CTCATTCCAATTACCATTTTACGGGGAGGTAGTGACAATA
Shen-B1	TGGTAATTGGAATGAGTTTTACCAGACTTGCCCTCCAATG
Shen-F2	ATTCCAATTACCAGACTTTTACGGGGAGGTAGTGACAATA
Shen-B2	TAATTGGAATGAGTACTTTTACCAGACTTGCCCTCCAATG
Shen-R-LF	BHQ1-GACTGAATT-JOE
Shen-X-LF	BHQ1-GACTCACTA-FAM
Shen-LB	AATCTAAATCCCTTAACG

**Table 2:** Experimental material information.

Number	Scientific Name	Source	Number	Scientific Name	Source
pg1	*P. ginseng*	White Mountain, Jilin, China	pq5	*P. quinquefolium*	America
pg2	*P. ginseng*	Tonghua, Jilin, China	Pq6	*P. quinquefolium*	Bozhou, Anhui, China
pg3	*P. ginseng*	Jixi, Heilongjiang, China	pq7	*P. quinquefolium*	Bozhou, Anhui, China
pg4	*P. ginseng*	Bozhou, Anhui, China	pq8	*P. quinquefolium*	Tonghua, Jilin, China
pg5	*P. ginseng*	Yanbian, Jilin, China	pq9	*P. quinquefolium*	Tonghua, Jilin, China
pg6	*P. ginseng*	America	pq10	*P. quinquefolium*	Yanbian, Jilin, China
pq1	*P. quinquefolium*	White Mountain, Jilin, China	pq11	*P. quinquefolium*	Fushun, Liaoning, China
pq2	*P. quinquefolium*	White Mountain, Jilin, China	Pg-S	*P. ginseng*	White Mountain, Jilin, China
pq3	*P. quinquefolium*	America	Pq-S	*P. quinquefolium*	White Mountain, Jilin, China
pq4	*P. quinquefolium*	Canada			

## Data Availability

The data presented in this study are available upon request from the corresponding author.

## Supplementary Materials

The following supporting information can be downloaded at: https://www.mdpi.com/article/10.3390/molecules28196872/s1, Figure S1: Mapping Proofman-LMITA primers for *P. quinquefolium* and *P. ginseng* on target sequences.

## Sample Availability

Samples of the compounds are not available from the authors.
