# Supplementary material for: Rapid Discrimination of Panax quinquefolium and Panax ginseng Using the Proofman-Duplex-LMTIA Technique
Source: Molecules. 2023 Sep 29;28(19):6872. doi: 10.3390/molecules28196872 (PMC10574230; doi:10.3390/molecules28196872)
Supplement: Supplementary file 1 [file molecules-28-06872-s001.zip › molecules-2598961-supplementary.pdf]

Supplementary File

Target sequence of *P. ginseng*

5'-ACGGGGAGGTAGTGACAATAAATAACAATACCGGGCTGATTCAGTCTGG*TAATTG*-3'
3'-TGCCCCTCCATCACTGTTATTTATTGTTATGGCCCGACTAAGTCAGACCATTAAC-5'
5'-*GAATGAGTAC*AATCTAAATCCCTTAACGAGGATCCATTGGAGGGCAAGTCTGGT-3'
3'-CTTACTCATGTTAGATTTAGGGAATTGCTCCTAGGTAACCTCCCGTTCAGACCA-5'

Target sequence of *P. quinquefolium*

5'-ACGGGGAGGTAGTGACAATAAATAACAATACCGGGCTCAGTGAGTCTGG*TAATTG*-3'
3'-TGCCCCTCCATCACTGTTATTTATTGTTATGGCCCGAGTCACTCAGACCATTAAC-5'
5'-*GAATGAGTAC*AATCTAAATCCCTTAACGAGGATCCATTGGAGGGCAAGTCTGGT-3'
3'-CTTACTCATGTTAGATTTAGGGAATTGCTCCTAGGTAACCTCCCGTTCAGACCA-5'

Primer Name	Sequence (5' to 3')
Shen-F1	CTCATTCCAATTACCATTTTACGGGGAGGTAGTGACAATA
Shen-B1	TGGTAATTGGAATGAGTTTTACCAGACTTGCCCTCCAATG
Shen-F2	ATTCCAATTACCAGACTTTTACGGGGAGGTAGTGACAATA
Shen-B2	*TAATTGGAATGAGTAC*TTTTACCAGACTTGCCCTCCAATG
Shen-R-LF	BHQ1-GACTGAATT-JOE
Shen-X-LF	BHQ1-GACTCACTA-FAM
Shen-LB	AATCTAAATCCCTTAACG

Figure S1. Mapping Proofman-LMITA primers for *P. quinquefolium* and *P. ginseng* on target sequences.
